# Supplementary figures and images for: In silico analysis and in planta production of recombinant ccl21/IL1β protein and characterization of its in vitro anti-tumor and immunogenic activity
Source: PLoS One. 2022 Aug 29;17(8):e0261101. doi: 10.1371/journal.pone.0261101 (PMC9423642; doi:10.1371/journal.pone.0261101)

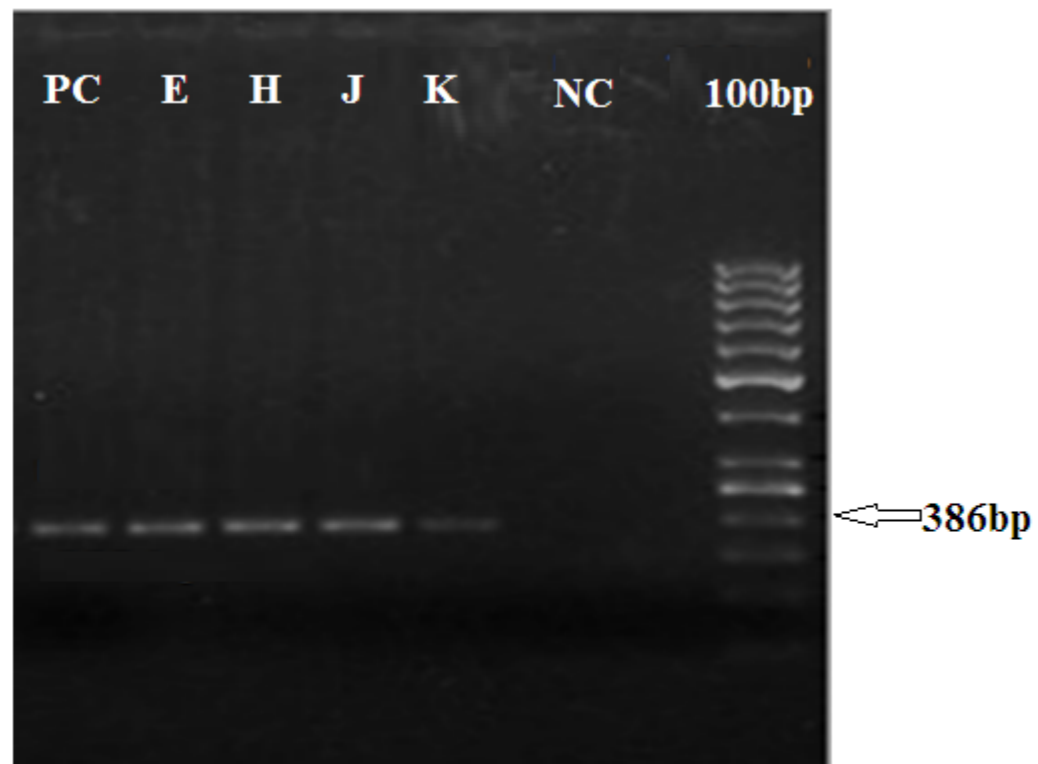

الف

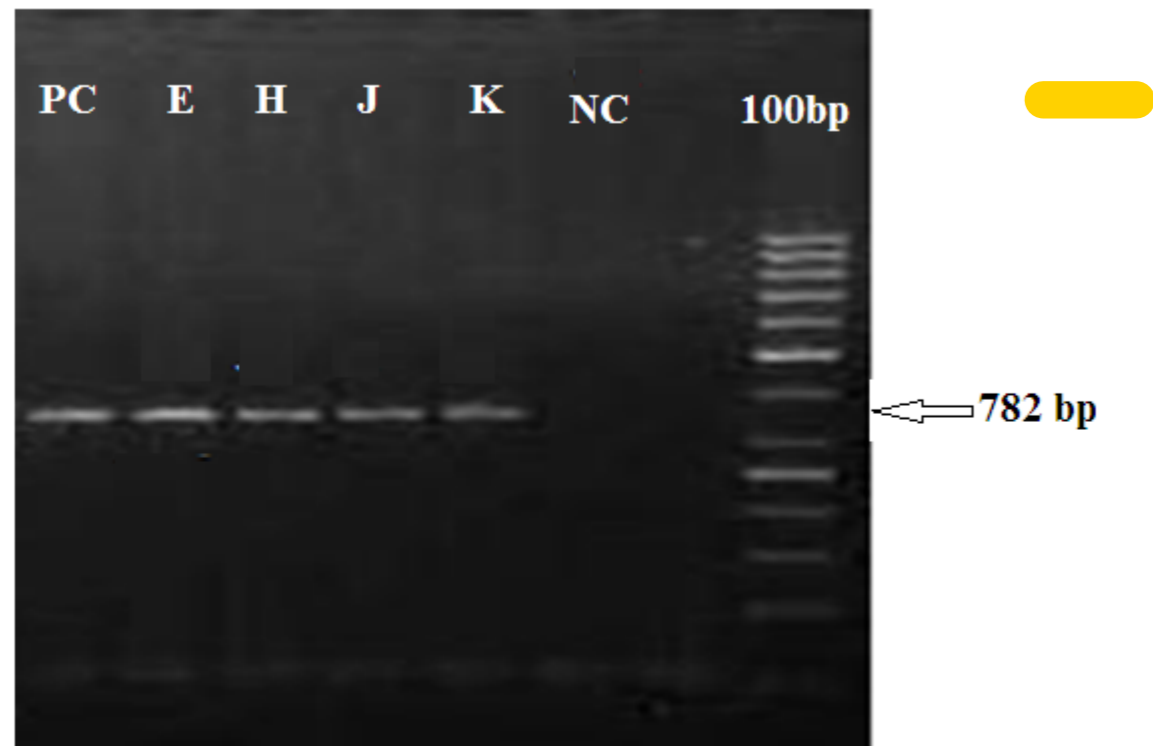

ب

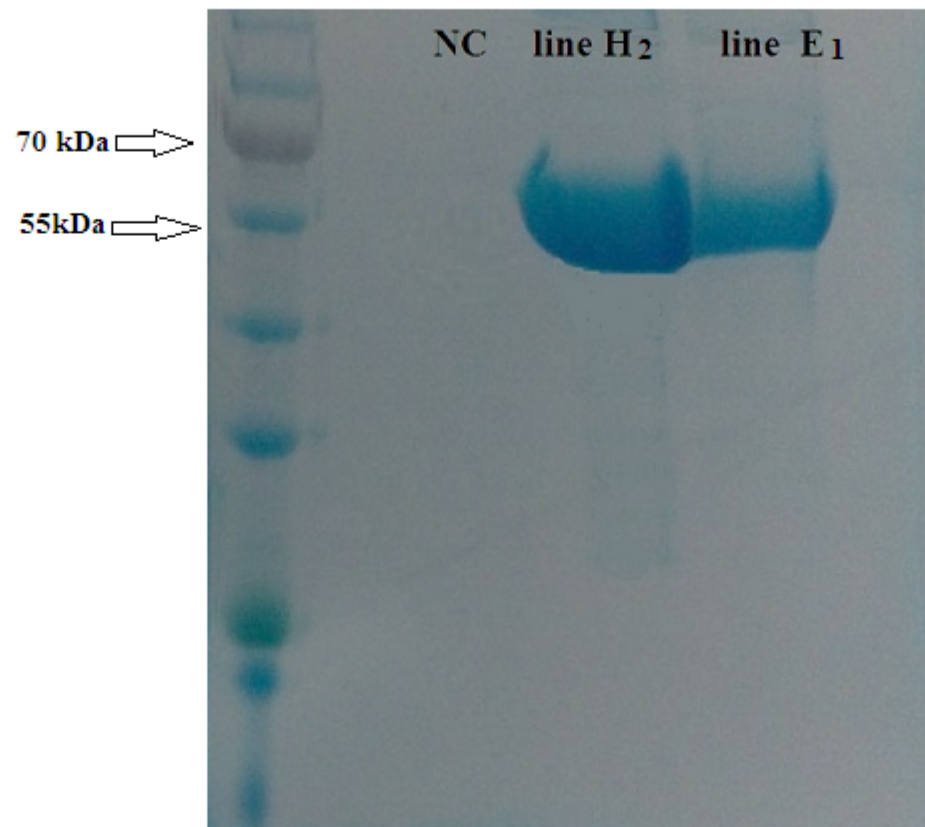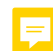

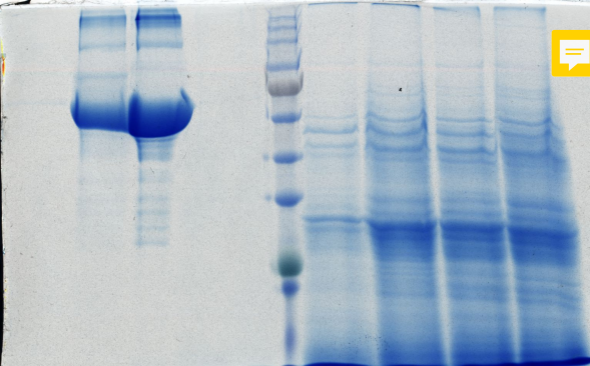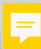

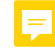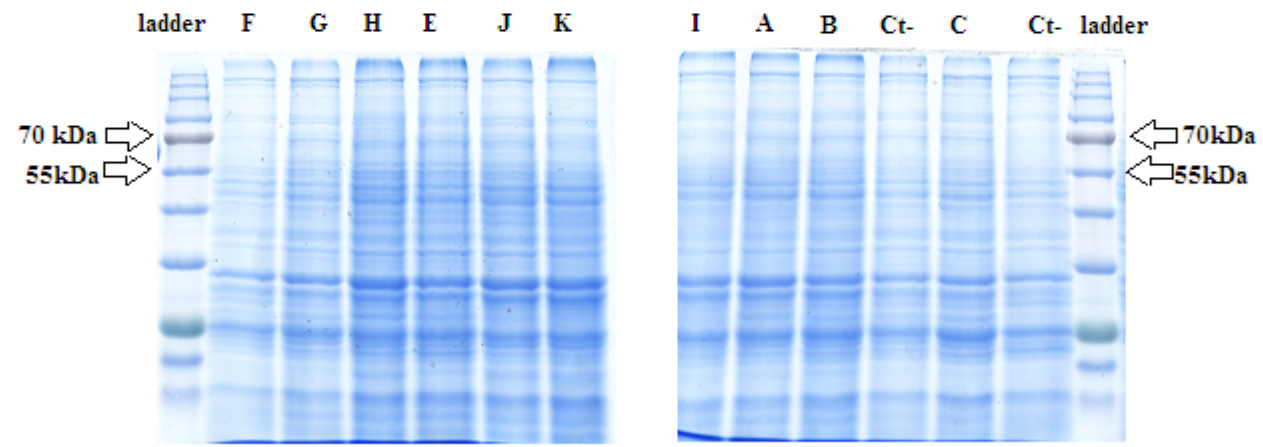

**elution 1**

**wash1**

**wash2**

**elution2**

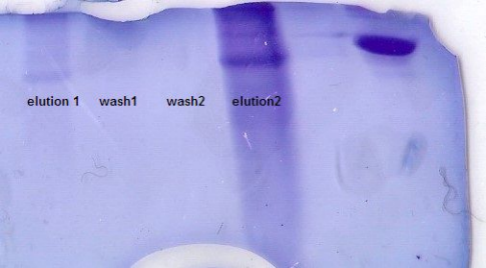

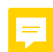

**A**

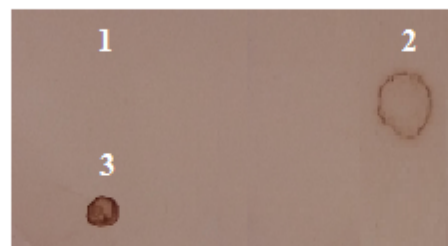

**B**

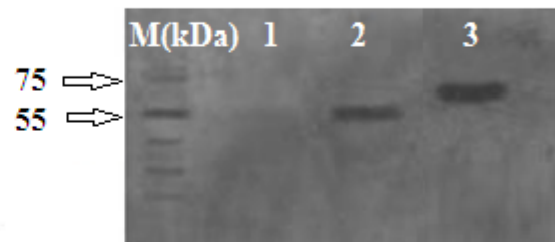

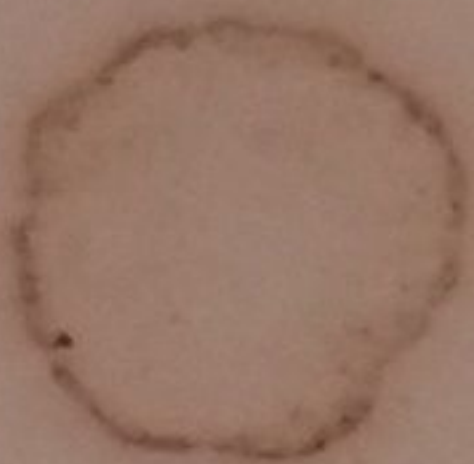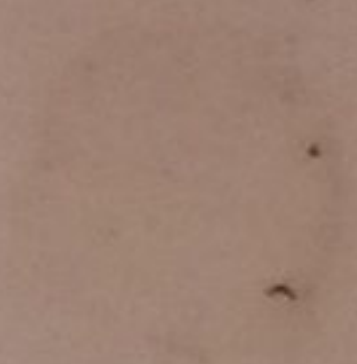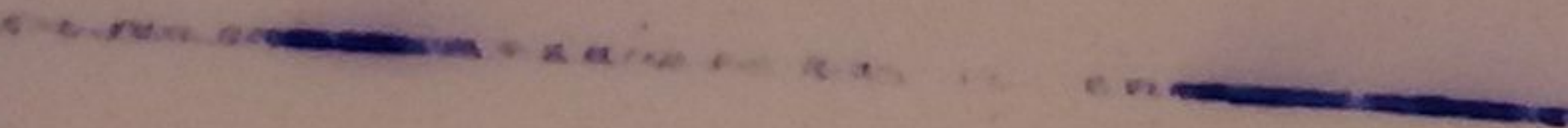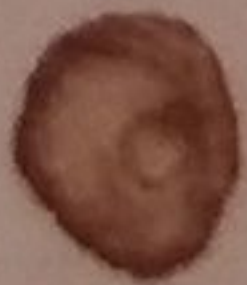

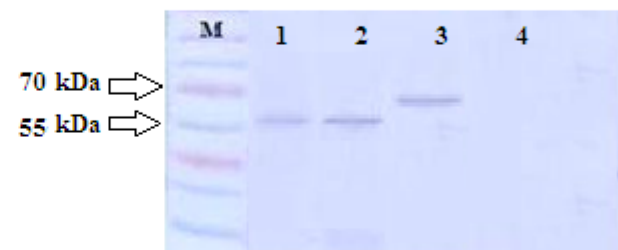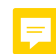

H2

E1

CCI21

54kDa

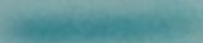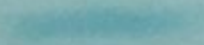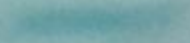

Supplement: S1 Raw image — (PDF) [file pone.0261101.s001.pdf]
