# Supplementary material for: In silico analysis and in planta production of recombinant ccl21/IL1β protein and characterization of its in vitro anti-tumor and immunogenic activity
Source: PLoS One. 2022 Aug 29;17(8):e0261101. doi: 10.1371/journal.pone.0261101 (PMC9423642; doi:10.1371/journal.pone.0261101)
Supplement: Attachment — Submitted filename: Reviewer.docx [file pone.0261101.s003.docx]

Reviewer #1

Dear Reviewer Thanks a lot for all of your comments, I have noticed point by point and highlight them in manuscript.

Best Regard

Maria Beihaghi

Reviewer #2:

The manuscripts represent an interesting piece of work which in principle is publishable. However, it requires significant modification in order to make it suitable for publication. Therefore, my recommendation is MAJOR REVISION. After the authors have completed these revisions, the new manuscript should undergo further peer review.
1- I guess my main concern about the manuscript is that in no place has the sequence of the chimeric CCL21/IL1beta protein spelt out. CCL21 interacts with its receptor through both the the N-terminus, and the main sequence whilst it’s C-terminus is mostly responsible for interaction with glucosaminoglycans on the surface of endothelial cells. Depending on where the IL1beta sequence is inserted, these interactions can significantly affect the molecular interactions of the peptide with its receptor, CCR7 (which is the point of the paper). I presume the purpose of the molecular modelling was to demonstrate that these interactions still work, although that is by no means clear.

Dear reviewer, Thank a lot you for your consideration.

As mentioned in your comment, I will add this paragraph in manuscript (2.1.1 Construction of Expression Cassette) and highlight it;

DNA sequences encoding human CCL21 (Accession No: [O00585](http://www.uniprot.org/uniprot/O00585)) had a DCCL motif, a domain binding to the CCR7 receptor and VQGEESNDK sequence; a part of selected epitopes of IL-1β is involved in inflammatory and immune responses and has high adjuvant effects, were designed as the principal part of the expression cassette. The designed expression cassette included Kozak consensus sequence as the plant-specific ribosome binding site (GCCACC). The CCL21 and VQGEESNDK amino sequences of human IL-1 beta were linked together by EAAAK sequence as beta-defensin linker that reduces interaction with other recombinant protein domains. The SEKDEL signal sequence - for effectual agglomeration of the recombinant protein in the endoplasmic reticulum (ER) - and 6xHis tags for purification of the gene construct were also linked by RVLAEA sequence as HIV protease linker respectively. Recognition sites of *Bam*HI and *SacI* restriction enzymes were added to the 5' and 3' ends of the synthetic gene, respectively. AUG and UAA were also added to the construct's 5' and 3' ends as start and stop codons, respectively. The terminal part of the chimeric gene was optimized based on the codon usage pattern of tobacco according to the Codon Usage Tabulated from GenBank (CUTG) website (http://www.kazusa.or.jp/codon/)

So we designed and synthesized a codon-optimized ccl21/IL1β gene from human genes for simultaneous activation of T and B cell lymphocytes, macrophages and neutrophils required to transfer protein to the target tissue.CCL21 is a Chemokine that controlled cell trafficking and involved in numerous pathologic and inflammatory conditions and it is endocytosed with its receptor (CCR7) .As it illustrate in lines13-15 and 44- 52 ; CCR7 receptor plays an important role in the immune balance and specifically the CC receptor7 and its ligands play an important role in the transport of T lymphocytes and antigen-presenting cells such as dendritic cells (DCs) to the lymph nodes (LNs).

**Figure .** Schematic diagram of the plant expression system construct used for agrobacterum transformation

I am also unclear about the size of the protein. On line 452, the authors state that they expected a MW of 55kDa (although they don’t explain why) which is quite large for a cytokine (CCL21 and IL1b have MW of 12.5 and 17.5 kDa respectively). However, figure 1B doesn’t look that different from the CCL21 WT. How is that possible? (see also comment 10)

As mentioned in line 364 Molecular mass (Mw), isoelectric point (pI) and solubility (%) of the recombinant protein were estimated to be 14,884.5504 Da, 9.377, and 0.8434, respectively by SOLpro server (<http://scratch.proteomics.ics.uci.edu/explanation.html#SOLpro>).

Also in line 476 ). In western blot analysis, the recombinant protein with an estimated molecular mass of 55 kDa was successfully detected under reducing conditions (Figure 3C). So results showed that; after purification, the recombinant protein did not migrate at the predicted molecular weight of 15.5 kDa under denaturing conditions, suggesting that the protein's mobility under denaturing conditions was influenced by its biochemical properties . So the purified recombinant protein was eluted as a single peak corresponding to a molecular weight of 55 kDa, suggesting that the protein might exist in a dimeric state in solution. Also as shown in Recombinant Human CCL21/6Ckine as commercial CCL21 (Cat No: 966-6C), The predicted Molecular mass is 38.3 kDa but under reducing condition the molecular mass of commercial CCL21 was about 65 kDa.

2- That omission, is only one example of the wider issue with the manuscript. It is highly unfocused and contained on occasion superfluous information, and on other occasions not enough information. For example, section 2.1.2 doesn’t really add anything to the manuscript. On the other hand in line 379-380 the authors state one of the 10 models generated was used without mentioning what the selection criteria was.

Dear reviewer , Thanks for the points you mentioned; I check it and just add this sentences in lines 399-400 ;Molecular modeling was generated by Modeler 9.24 and one model selected as the most suitable for the rest of the project (Figure 1B).
3- There are quite a large number of errors and omission in the manuscript. These are far too many for me to list but as an example:
4- In line 459, the authors mention the sequence VQEESNDK from IL1b and mass is given as 947.1 Da, which is correct for that sequence. This is used as proof of inclusion of IL1b. However, according to the authors themselves in lines 95 and 363 (and also according to Uniprot) the correct sequence is V(162)QGEESNDK(171) with a mass of 1004 Da. Can the authors explain this discrepancy?

Dear reviewer , Thanks for the points you mentioned; I check it and you told true , I correct this paragraph in lines 516-520 ;

Mascot analysis demonstrated that CSIPAILFLPR and QGEESNDK sequences are part of human CCL21 and ILIβ with molecular weights of 1229.55 Da and 947.1 Da, respectively, whereas no tobacco sequences were detected. The average protein sequence coverage was 12%, which confirmed recombinant CCL21/IL1β as the target protein that was correctly expressed and purified from transgenic lines.

**IL1β**

**CCL21**

The MALDI-TOF/TOF results were analyzed using Mascot software. Searches used the concatenated Mascot Generic Format (mgf) file. Mascot software identified two peptides matched to the expected CCL21/IL-1β sequences with a high score. Mascot analysis demonstrated that CSIPAILFLPR and QGEESNDK amino acids are human sequences of CCL21 and IL-Iβ with 1229.55 Da and 947.1 Da respectively, whereas no protein sequence for tobacco plant was detected. The average protein sequence coverage was 12%, which confirmed CCL21/IL-1β recombinant protein as a target protein that was correctly expressed and purified from transgenic lines.

5- Line 363 “Protein sequences encoding human CCL21 used in this structure have a DCCL motif…”

Yes, your revise was true. As mentioned in line 95 Protein sequences encoding human CCL21 (Accession No: [O00585](http://www.uniprot.org/uniprot/O00585)) had a DCCL motif, So I explain these sequences;

In an exemplary embodiment, each of the truncated chemokines may include a DCCL motif, a putative receptor binding cleft, and a putative glycosaminoglycan binding	site.

The CCL21 sequences selected epitopes had a DCCL motif, a domain binding to the CCR7 receptor, and a Pan HLA DR-binding epitope (PADRE) peptide sequence and a putative glycosaminoglycan binding site that covers more than 90% of the HLA alleles.

This below sequence

MSDGGAQDCCLKYSQRKIPAKVVRSYRKQEPSLGCSIPAILFLPRKRSQAELCADPKELWVQQLMQHLDKTPSPQKPAQGCRKDRGASKTGKKGKGSKGCKRTERSQTPKGPVQGEESNDKSEKDELHHHHHH

KELWVQQLMQHL (putative glycosaminoglycan (GAG) binding site)

DCCL (a domain binding to the CCR7 receptor)

WVQQLMQHLD (putative receptor binding site)

CCLKY (putative receptor binding site)

IPAKVV (putative receptor binding site)

6- Line 365 “Since their IC50 is less than 50” (no unit given) etc etc etc

Yes I attend to this point
7- I recommend the authors do not use “CCR7+” when refer to CCR7 expressing immortalised cell line, MCF-7. The terminology is widely used for dendritic cells and T cells where gain of expression is associated with gain of functional capabilities of the cells. For immortalised cell lines, “+” is often used in the context of knock in, so it’s use can be confusing.

Yes, your revise was true so all points you said were corrected

8- Related to the above, although there are no strict guidelines for this journal, I strongly recommend to the editor that the authors should provide an STR based certificate of authentication for the MCF-7 cell line used in this study. (Even though there is no doubt MCF-7 do express CCR7, see <https://pubmed.ncbi.nlm.nih.gov/21151474/>).

Thanks for your attention, yes we want to demonstrate that if this recombinant protein can be act like commercial CCL21 or not? So this study aimed to investigate the expression of *ccl21/IL1β* as recombinant protein in transgenic lines

All assays such as; ***In Vitro* wound assay, MTT cytotoxicity assay, Migration of PBMC cells (Chemotaxis Assay)** MCF-7 Human breast cancer cells were treated with different concentrations of CCL21/IL1β recombinant protein, non-recombinant protein as the negative control, CCL21 commercial protein as the positive control, and also DMEM high glucose culture medium as the control,
9- Generally speaking the quality of the figures is very poor. The link for reviewers did not contain the original figures.

Thanks for your attention, I will send you some original figures also some figures have ben changed.

10- In the manuscript, the in silico data for binding of CCL21-IL1b chimera with CCR7 is purely visual. Obviously this is not sufficient information to support the key hypothesis of the manuscript, so authors need to provide more information on the interactions between the chimera and receptor at a molecular level.

Dear reviewer

Stability and binding affinity of CCL21/IL1β protein and CCR7 receptor were examined through *in silico* analyses . So result of Molecular modeling and dynamics simulation of the recombinant protein and Molecular docking and simulation of multi-epitope protein with the CCR7 receptor was mentioned in paragraph 3.1.3 and 3.1.4 . Also figure 1E&G show the interactions between the chimera and receptor at a molecular level.

Best Regard

Maria Beihaghi
