# Supplementary material for: In silico analysis and in planta production of recombinant ccl21/IL1β protein and characterization of its in vitro anti-tumor and immunogenic activity
Source: PLoS One. 2022 Aug 29;17(8):e0261101. doi: 10.1371/journal.pone.0261101 (PMC9423642; doi:10.1371/journal.pone.0261101)
Supplement: Attachment — Submitted filename: Reviewer.docx [file pone.0261101.s004.docx]

Reviewer #1

Dear Reviewer Thanks a lot for all of your comments, But most of your comments was changed in manuscript track changes file that have been sent in revise submission file before. So I have noticed point by point and add some paragraph and highlight them in manuscript track changes file, Also the other comment was mentioned and noticed in PONE-D-21-35241_reviewer_comments-3.pdf file.

1- I assume there is more than one, so this should be specified.

As mentioned in your comment, There are five phases for wound healing; hemostasis, inflammation, cellular migration and proliferation, protein synthesis and wound contraction, and remodeling . However, mainly only three phases, inflammatory, proliferative, and remodeling are presented due to the overlap of phases. So lines 21- 23 in abstract ,pointed to proliferative phase (Potential activity of purified CCL21/IL1β in stimulating the proliferation and migration of MCF7CCR7+ cancer ).and I will add above paragraph in manuscript (2.9 In Vitro wound healing assay) and highlight it.

**3.7 Migration of the cancer cell line into the scratch**

Migration rate of MCF7 cells was estimated in three groups: a group with commercial CCL21 antigen as the positive control, the medium culture group without plant protein extract as the negative control, and the purified protein extract from transgenic tobacco leaves as the third group. Medium concentrations of the extract (7.5 µg/ml) induced migration of the cancer cells and closure of the scratch, and the percentage of closure was calculated using Image J. software. When assessed 24 hours post-treatment, the two control groups had migrated by 22% to scratch. After 48 hours, the scratch closure was 88%. Surprisingly, 72 hours following the same treatment, the scratch was almost completely closed. However, 24 hours after treatment with the same concentration of the purified protein extract of transgenic leaves, no evident migration was detected for the cells; but 15% and 28% scratch closure occurred after 48 and 72 hours, respectively. The results suggest that the extraction of plant recombinant protein was negatively related to the migration rate of the cells (Figure 5).

**Explain agarose assay**

**2.11 Under agarose gel assay**

Under agarose gel assay was used to investigate the mechanism of CCl21 impact on T-cell migration because chemokines are cytokines involved in leukocyte migration; Chemotaxis is the primary mechanism by which cell movements are directed within multicellular organisms, and it is a major component of embryonic development, wound healing, and immune responses.

| **chemotaxis percent** | **chemotaxis rate**  **(A-B)** | **The number of cells in the well contains cells and non-absorbent material**  **(B)** | **The number of cells in the well contains cells and absorbent material**  **(A)** | **adsorbent substance** |
| --- | --- | --- | --- | --- |
| 90 | 153 | 17 | 170 | **CCL21 Commercial Protein**  (positive control as chemokine) |
| **91** | 126 | 12 | 138 | **Recombinant Protein CCL21** |
| 89 | 141 | 17 | 158 | **FBS10%**  (positive control as chemotaxis) |

Chemotaxis involves a complex cascade of events--formation of signaling complexes, receptor polarization, adhesion molecule activation, and cytoskeletal reorganization

**Table 3.**The approximate count of the migrated cells and the percentage of chemotaxis according to Figure 8 A to D.

“The number of cells in the well contains cells and absorbent material” – “The number of cells in the well containing cells and non-absorbent material” = chemotaxis rate

The number of cells in the well containing cells and absorbent material/chemotaxis rate) × 100 = the percentage of chomotoxicity (Mousseau et al., 2007).

Best Regard

Maria Beihaghi
