# Supplementary material for: In silico analysis and in planta production of recombinant ccl21/IL1β protein and characterization of its in vitro anti-tumor and immunogenic activity
Source: PLoS One. 2022 Aug 29;17(8):e0261101. doi: 10.1371/journal.pone.0261101 (PMC9423642; doi:10.1371/journal.pone.0261101)
Supplement: Attachment — Submitted filename: Reviewer.docx [file pone.0261101.s005.docx]

Reviewer #1

Dear Reviewer Thanks a lot for all of your comments, I have noticed point by point and highlight them in manuscript.

1- the abstract has to be self-explanatory. Therefore, responding that, for example, the wound healing method, is explained in the main text appears to be insufficient.

Dear Reviewer Thanks a lot for your comments, Several experiments have been carried out in this project and considering that the maximum number of words that must be used is 250 words, so unfortunately we can't explain more in abstract.

2- the description of the statistical analysis is insufficient, e.g. which tests were used to establish normality of the data before applying Duncan’s test?

The data was classified into two categories: recombinant and non-recombined. In each category, there are four independent groups with concentrations of 2.5, 5, 7.5 and 10µg/ml and a control group. In each group, the experiment was repeated three times. In addition, the mentioned structure has been implemented in three periods of 24, 48 and 72 hours. By performing the Kolmogorov-Smirinov test on the obtained data, we conclude that the data has normal characteristics. In each time intervals, the concentrations of 2.5, 5, 7.5 and 10 will be compared with the control group, by using one-way analysis of variance and Scuffle’s within-group test for recombinant and non-recombinant categories. Also, the paired T-test was used to compare recombinant and non-recombined groups. These tests are done by using SPSS 26.In investigating the hypothesis of "Significant difference between the concentrations (7.5 with other concentrations) in the time period of 72 hours", we performed the ANOVA statistical test with Scheffe's within-group test for recombinant, non-recombinant and commercial proteins separately. Since the calculated P-Value for the comparisons of (2.5 and 7.5), (5 and 7.5) and (10 and 7.5) was less than 0.05, so the hypothesis is accepted.

3- Why was the defensine linker used? The authors claim that this is explained in lines 31-57 (which is rather vague), but this linker is not mentioned at all in this section.

Dear reviewer, Thank a lot for your consideration. You tell right β-defensin adjuvant thoroughly basic 45 amino acids long peptide that behaves both as an antimicrobial agent and as an immunomodulator. So as mentioned in your comment, I noticed that I just use EAAAK as flexible linker so beta-defensin epitope wasn’t use in this construct and I have deleted beta-defensin in manuscript (2.1.1 Construction of Expression Cassette) line 100;

**Best Regard**

**Maria Beihaghi**
